# Supplementary material for: Specification of female germline by microRNA orchestrated auxin signaling in Arabidopsis
Source: Nat Commun. 2022 Nov 15;13:6960. doi: 10.1038/s41467-022-34723-6 (PMC9666636; doi:10.1038/s41467-022-34723-6)
Supplement: Supplementary file 1 — Supplementary Information [file 41467_2022_34723_MOESM1_ESM.pdf]

Supplementary Information – Huang et al, *Nature Communications*

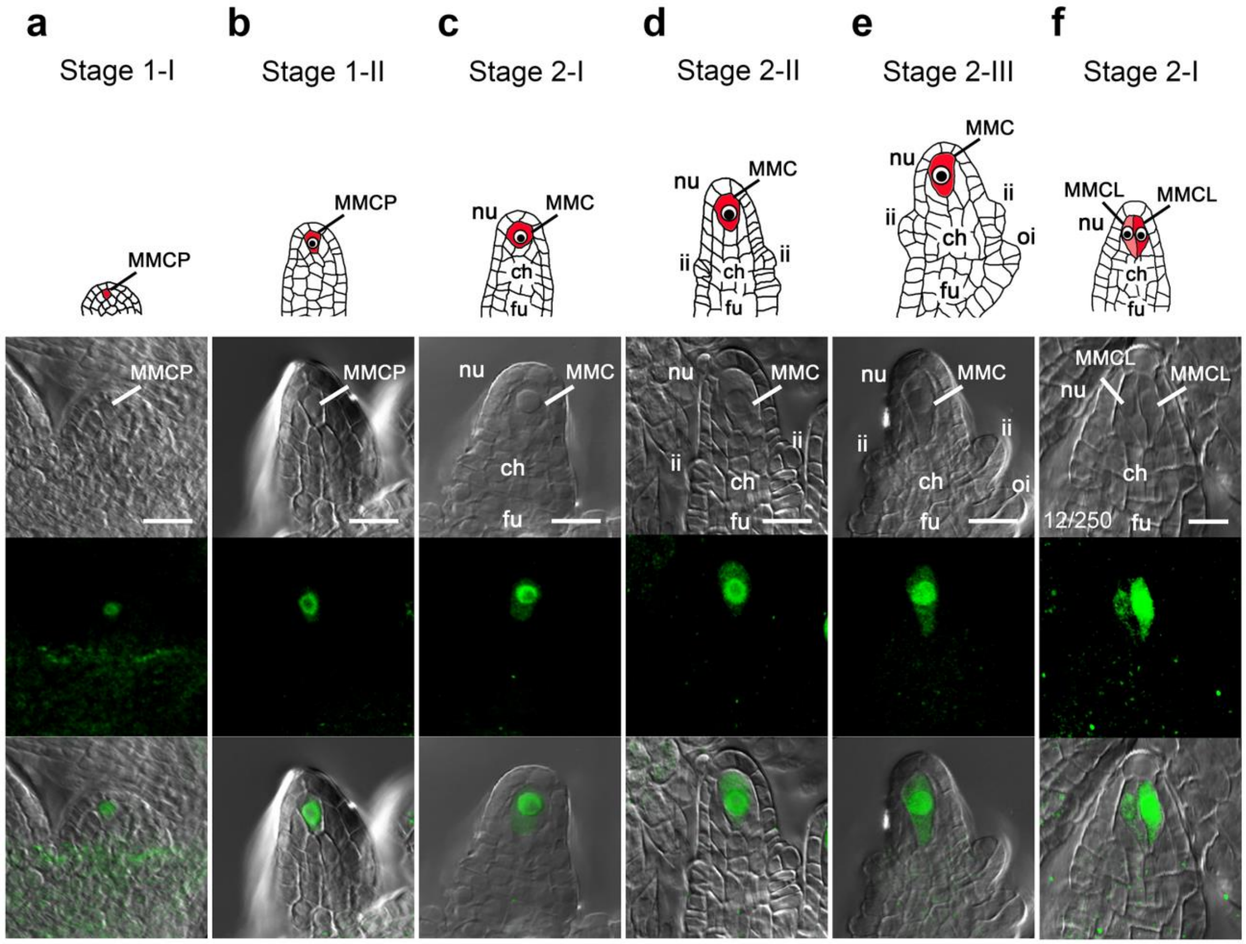

Supplementary Fig. 1 Defining MMC specification by the MMC molecular marker *pKNU::KNU-VENUS*.

a-**e**, MMC differentiation and expression of *pKNU::KNU-VENUS* in ovules at different stages. ch: chalaza, fu: funiculus, ii: inner integument, MMC: megaspore mother cell, MMCL: MMC-like cell, MMCP: megaspore mother cell precursor, nu: nucellus, and oi: outer integument. First row: Schematic diagrams showing MMC differentiation during ovule development at different stages. Second row: Differential interference contrast (DIC) images. Third row: Confocal images of ovules. Fourth row: Merged confocal and DIC images of images in second and third rows. **a**,

At stage 1-I, an ovule primordium appears as a dome structure along with the placenta. Cells underneath the epidermis of ovule primordium are not distinguishable. A weak signal of *pKNU::KNU-VENUS* is present in the MMCP. **b**, At stage 1-II, the ovule primordium continues to expand and gives rise to an elongate protrusion. The MMCP at the distal end of nucellus is still morphologically similar to somatic cells surrounding it. A moderate signal of *pKNU::KNU-VENUS* is observed in the MMCP. **c**, At stage 2-I, the MMC becomes distinct from somatic cells surrounding it, indicated by large cell and nucleus size. A strong signal of *pKNU::KNU-VENUS* is found in the MMC. **d**, At stage 2-II, the inner integuments (ii) are initiated. The size of MMC and its nucleus continues to enlarge. A strong signal of *pKNU::KNU-VENUS* is present in the MMC. **e**, At stage 2-III, the outer integuments (oi) are initiated. The MMC is expanded along the distal-proximal axis. A strong signal of *pKNU::KNU-VENUS* is detected in the MMC. **f**, A WT ovule with 2 MMCLs at stage 2-I. Two cells express *pKNU::KNU-VENUS*, although the signal in one cell is weaker than that in the other cell. The number (12/250) in the rightmost panel in the second row denotes the frequency of two MMCLs. Experiments were repeated three times with similar results. Scale bars, 10 µm.

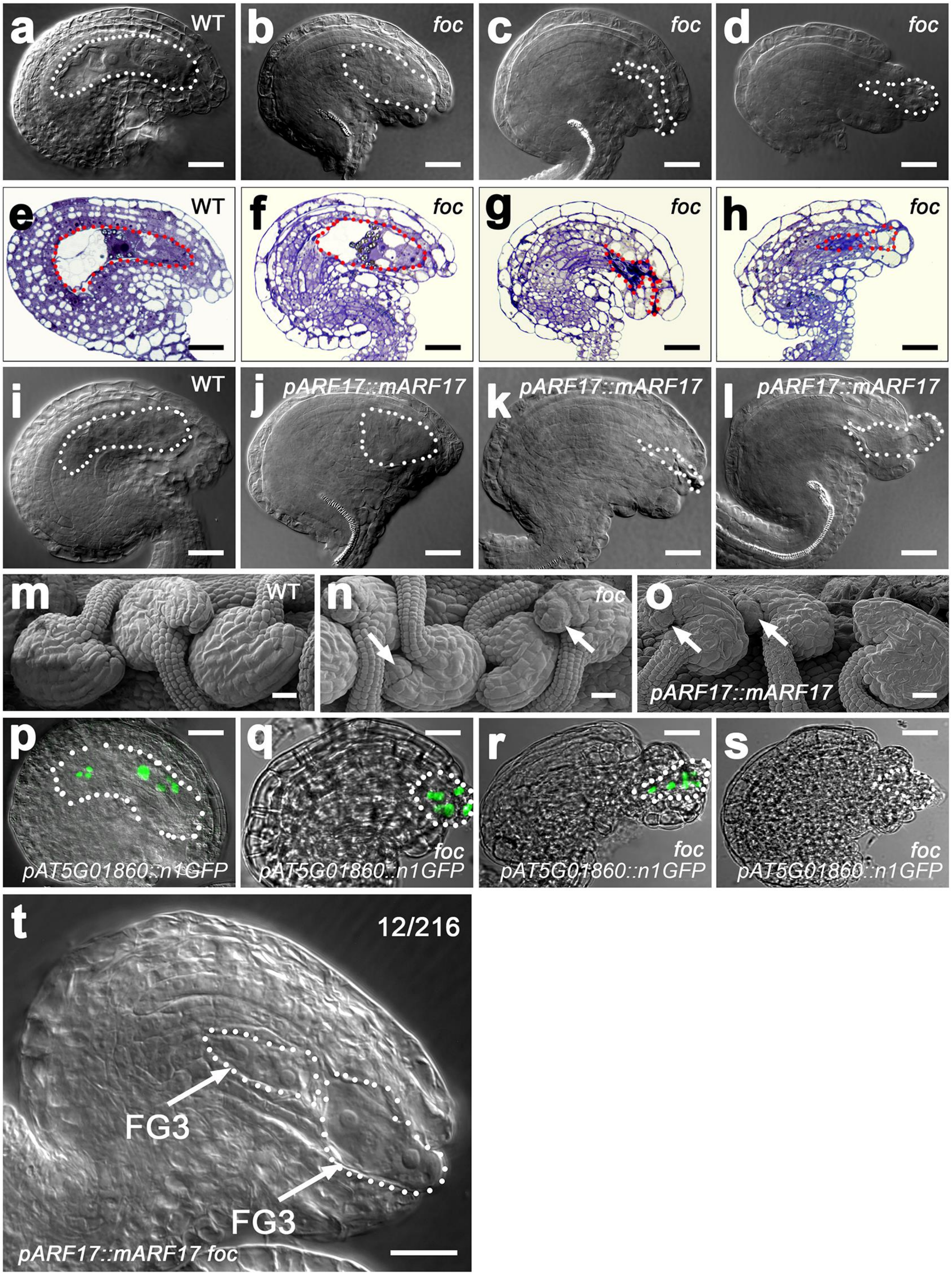
a
WT
b
foc
c
foc
d
foc
e
WT
f
foc
g
foc
h
foc
i
WT
j
pARF17::mARF17
k
pARF17::mARF17
l
pARF17::mARF17
m
WT
n
foc
o
pARF17::mARF17
p
pAT5G01860::n1GFP
q
foc
pAT5G01860::n1GFP
r
foc
pAT5G01860::n1GFP
s
foc
pAT5G01860::n1GFP
t
12/216
FG3
FG3
pARF17::mARF17 foc

Supplementary Fig. 2 Defects of embryo sacs during later female gametophyte development in *foc* and *pARF17:mARF17* ovules.

a-**d**, DIC images showing embryo sacs (outlined by white dotted lines) in WT and *foc* ovules at stage FG6. **a**, A mature embryo sac in the WT ovule. **b**-**d**, Aberrant embryo sacs (84.4%, n = 262) in *foc* ovules. **b**, A short embryo. **c**, A collapsed embryo sac. **d**, A collapsed embryo sac that is not fully enclosed by integuments. Experiments were repeated three times with similar results. **e**-**h**, Semi-thin sections displaying embryo sacs (outlined by red dotted lines) in WT and *foc* ovules at stage FG6. **e**, A mature embryo sac in the WT ovule. **f**-**h**, Abnormal embryo sacs in *foc* ovules. **f**, A short embryo sac. **g**, A collapsed embryo sac. **h**, A collapsed embryo sac that is not fully enclosed by integuments. Experiments were repeated three times with similar results. **i**-**l**, DIC images showing embryo sacs (outlined by white dotted lines) in WT and *pARF17::mARF17* ovules at stage FG6. **i**, A mature embryo sac in the WT ovule. **j**-**l**, aberrant embryo sacs (78.6%, n = 285) in *pARF17::mARF17* ovules. **j**, A short embryo. **k**, A collapsed embryo sac. **l**, A collapsed embryo sac that is not fully enclosed by integuments. Experiments were repeated three times with similar results. **m**-**o**, Scanning electron microscope (SEM) images showing ovules at stage FG6 in siliques. **m**, Normal WT ovules. **n**, Unenclosed *foc* ovules (arrows). **o**, Unenclosed *pARF17::mARF17* ovules (arrows). Experiments were repeated twice with similar results. **p**-**s**, Merged confocal and DIC images showing embryo sacs (outlined by white dotted lines) using the embryo sac molecular marker *pAT5G01860::n1GFP* in WT and *pAT5G01860::n1GFP foc* ovules at stage FG6. **p**, Discrete GFP signals in the WT embryo sac. **q**-**s**, Aberrant signals in the *foc* background. **q**, A cluster of GFP signals close to the micropyle in a short embryo sac. **r**, A cluster of GFP signals close to the micropyle in a short embryo sac that is not fully covered by integuments. **s**, No GFP signal in a collapsed embryo sac. Experiments

were repeated three times with similar results. **t**, A DIC image showing two embryo sacs (enclosed by white dotted lines) with two nuclei at the FG3 stage in an *pARF17::mARF17-GFP foc* ovule. Experiments were repeated twice with similar results. Scale bars, 10 µm.

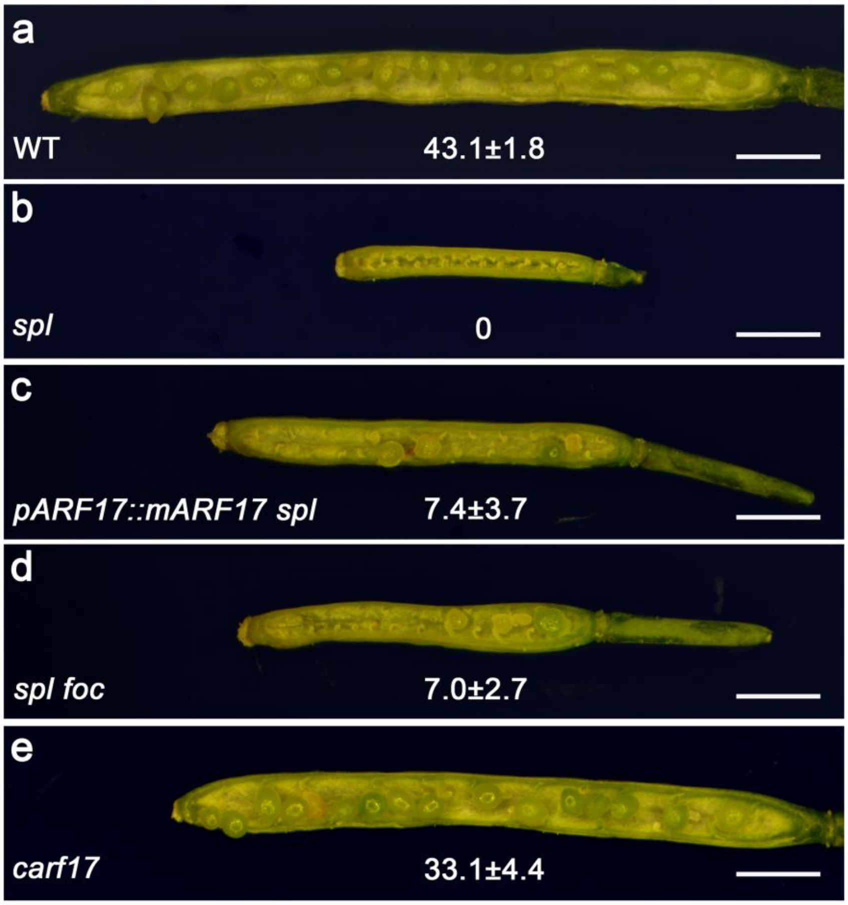

Supplementary Fig. 3 Examination of female fertility in *spl, pARF17::mARF17 spl, spl foc,* and *carf17* plants by manual pollination.

a-e, Opened siliques showing developing seeds resulting from manual pollination. **a**, WT. **b**, *spl*. **c**, *pARF17::mARF17 spl*. **d**, *spl foc*. **e**, *carf17*. The values are the mean ± SD of three independent experiments, each with fifteen siliques from three plants. Scale bars, 1 mm. Source data are provided as a Source data file.

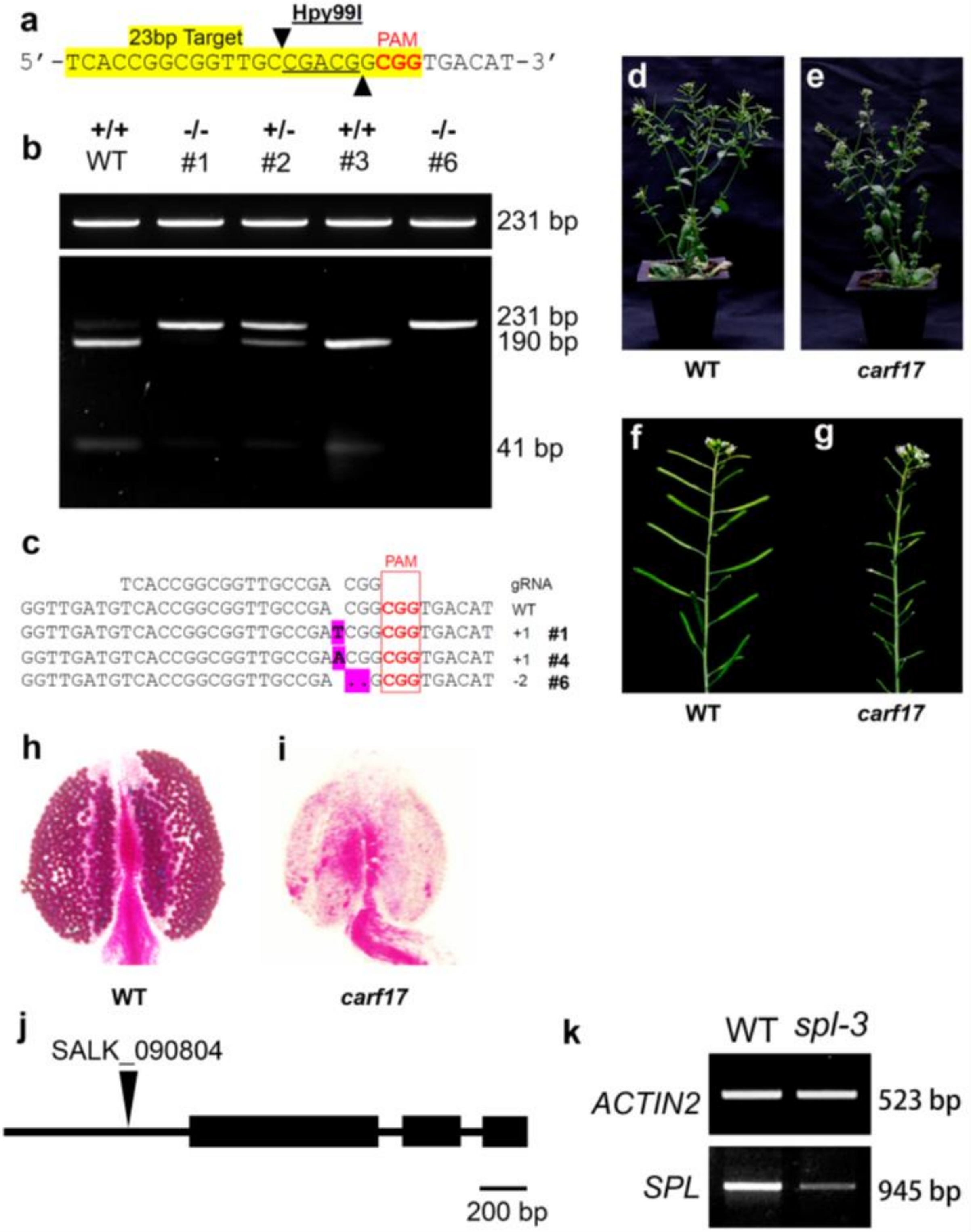
a
Hpy99I
23bp Target
PAM
5′-TCACCGGCGGTTGCCGACGGCGGTGACAT-3′
b
+/+ WT
-/- #1
+/- #2
+/+ #3
-/- #6
231 bp
231 bp
190 bp
41 bp
c
PAM
TCACCGGCGGTTGCCGA CGG gRNA
GGTTGATGTCACCGGCGGTTGCCGA CGGCGGTGACAT WT
GGTTGATGTCACCGGCGGTTGCCGATCGGCGGTGACAT +1 #1
GGTTGATGTCACCGGCGGTTGCCGAACGGCGGTGACAT +1 #4
GGTTGATGTCACCGGCGGTTGCCGA GCGGTGACAT -2 #6
d
e
WT
carf17
f
g
WT
carf17
h
i
WT
carf17
j
SALK_090804
200 bp
k
WT
spl-3
ACTIN2
523 bp
SPL
945 bp

Supplementary Fig. 4 Identification of *crispr-arf17* and *spl-3* mutants.

a-**i**, Identification of *crispr-arf17* (*carf17*) mutants. **a**, Sequence of the target site in the *ARF17* gene. The PAM and the Hpy99I cleavage sites are indicated. **b**, Mutation analysis by Hpy99I digestion of PCR fragments. The upper panel is PCR products from different lines without digestion. The lower panel shows the digested PCR products. The result shows that #1 and #6 are homozygous lines, and the #2 is a heterozygous line. The #3 is the WT. Experiments were repeated twice with similar results. Source data are provided as a Source data file. **c**, Alignment of sequences from PCR products amplified from mutants showing resistance to the Hpy99I digestion. **d**, **e**, Whole plants showing the normal growth of WT (**d**) and *carf17* (**e**) plants. **f**, **g**, Main inflorescences showing the normal fertility of WT (**f**) and sterility of *carf17* (**g**) plants. **h**, **i**, Pollen staining showing normal viable pollen grains in the WT anther (**h**), but no pollen grains in the *carf17* anther (**i**). **j**, **k**, Identification of the *spl-3* mutant. **j**, The schematic structure of the *SPL/NZZ* gene and the location of T-DNA insertion in the *SALK_090804* (*spl-3*) mutant. Solid boxes: exons. Arrowhead indicates that the T-DNA is inserted 383bp upstream of ATG in the *SPL/NZZ* promoter. **k**, RT-PCR result showing expression of *ACTIN2* (internal control) and *SPL/NZZ* in inflorescences from WT and *spl-3* plants. The *SPL/NZZ* gene expression is reduced in the *spl-3* inflorescence compared with that of WT. Experiments were repeated three times similar results. Source data are provided as a Source data file.

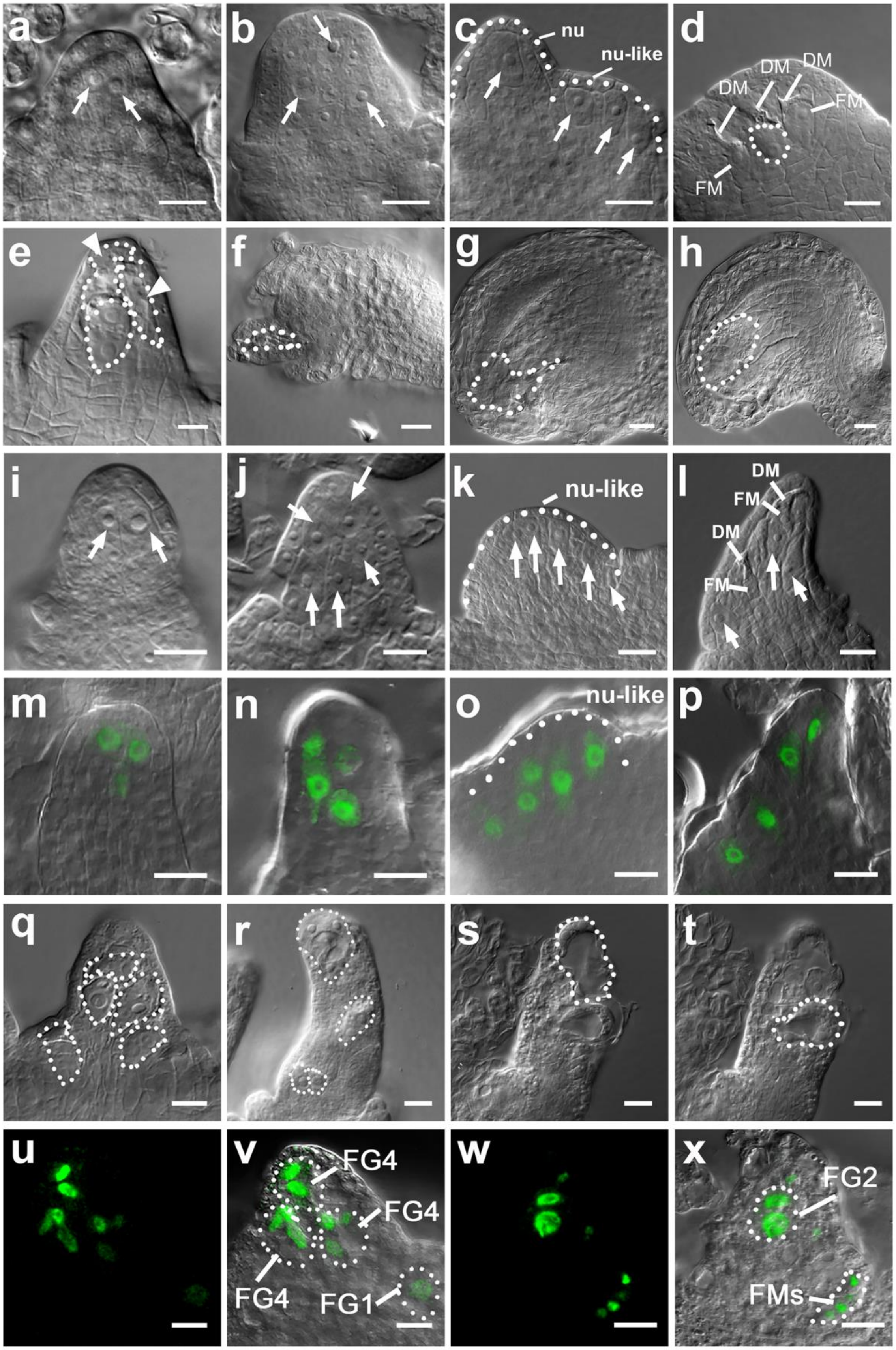
a
b
c
nu
nu-like
d
DM
DM
DM
FM
FM
e
f
g
h
i
j
k
nu-like
l
DM
FM
DM
FM
m
n
o
nu-like
p
q
r
s
t
u
v
FG4
FG4
FG4
FG1
w
x
FG2
FMs

Supplementary Fig. 5 Defects of MMC formation and embryo sac development in *pin1-5* and NPA-treated ovules.

a-**c**, DIC images showing supernumerary MMCLs (Arrows, 35.0%, n = 160) in *pin1-5* ovules. **a**, Two MMCLs. **b**, Three MMCLs. **c**, One MMCL in the nucellus (nu) and three MMCLs in a nucellus-like (nu-like) structure. White dotted lines indicate a nucellus and a nucellus-like structure. **d**-**h**, DIC images showing abnormal embryo sacs (outlined by white dotted lines) in *pin1-5* ovules. **d**, An ovule with two functional megaspores (FM), one embryo sac, and three degenerated megaspores (DM). **e**, Two collapsed embryo sacs (arrowheads) and one embryo sac with two nuclei. **f,** An uncovered and collapsed embryo sac. **g**, A collapsed embryo sac. **h**, A short embryo sac. **i**-**l**, DIC images showing supernumerary MMCLs (Arrows, 62.6%, n = 380) in ovules after NPA treatment. **i**, Two MMCLs. **j**, Five MMCLs. **k**, Five MMCLs in a nucellus-like (nu-like) structure (white dotted lines). **l**, Three MMCLs (arrows) mixed with two functional megaspores (FM). DM: degenerated megaspores. **m**-**p**, Merged confocal and DIC images showing NPA-treated ovules expressing *pKNU::KNU-VENUS* marking the MMC fate. **m**, Two MMCLs. **n**, Four MMCLs. **o**, Five MMCLs in a nucellus-like (nu-like) structure (indicated by the white dotted line). **p**, Four MMCLs scattered in the nucellus from the distal end to the proximal end. **q**-**t**, DIC images showing irregular numbers of embryo sacs (outlined by white dotted lines, 39.8%, n = 211) in ovules after NPA treatment. **q**, Clustered embryo sacs. **r**, Relatively discrete embryo sacs with different numbers of nuclei. **s**, An embryo sac at FG4 stage (4 nuclei) at the distal end of nucellus. **t**, Another embryo sac at FG4 stage in the same ovule in (**s**). **u**-**x**, Confocal (**u**, **w**) and merged confocal and DIC (**v**, **x**) images showing NPA-treated ovules expressing *pAT5G01860::n1GFP* marking embryo sacs [outlined by white dotted lines in (**v**, **x**)]. **u**, **v**, Three clustered embryo sacs at stage FG4 and one embryo sac at stage FG1 (one

nucleus). **w**, **x**, Two discrete embryo sacs at the FG2 stage (2 nuclei) and the FM stage (FMs), respectively. Scale bars, 10 μm.

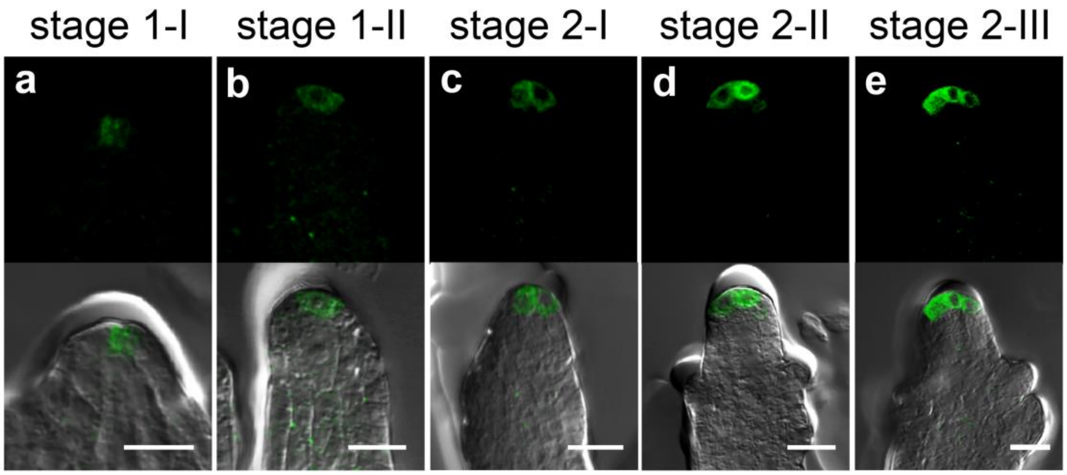

Supplementary Fig. 6 A single auxin maximum resides in one or a few cells at the apex of nucellus during MMC differentiation.

a-**e**, Confocal (top row) as well as merged confocal and DIC (bottom row) images showing a single auxin maximum at the apex of nucellus in ovules expressing *DR5rev::GFP*. **a**, **b**, Auxin maximum in one cell at stage 1-I (**a**) and stage 1-II (**b**). **c**, **d**, Auxin maximum in two cells at stages 2-I (**c**) and 2-II (**d**). **e**, Auxin maximum in three cells at stage 2-III. Scale bars, 10 µm. Experiments were repeated three times with similar results.

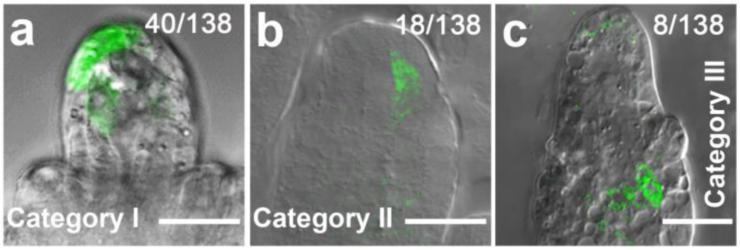

Supplementary Fig. 7 Abnormal number and location of auxin maxima in *DR5rev::GFP pEMS1::YUC1* ovules.

a-c, Merged confocal and DIC images showing auxin maximum alterations in *DR5rev::GFP pEMS1::YUC1* ovules. **a**, GFP signals showing expanded apical auxin maxima (Category I). **b**, The GFP signal displaying centrally shifted auxin maxima (Category II). **c**, GFP signals exhibiting basally shifted auxin maxima (Category III). Numbers in the panels denote frequencies of phenotypes shown. Experiments were repeated three times similar results. Scale bars, 10 µm.

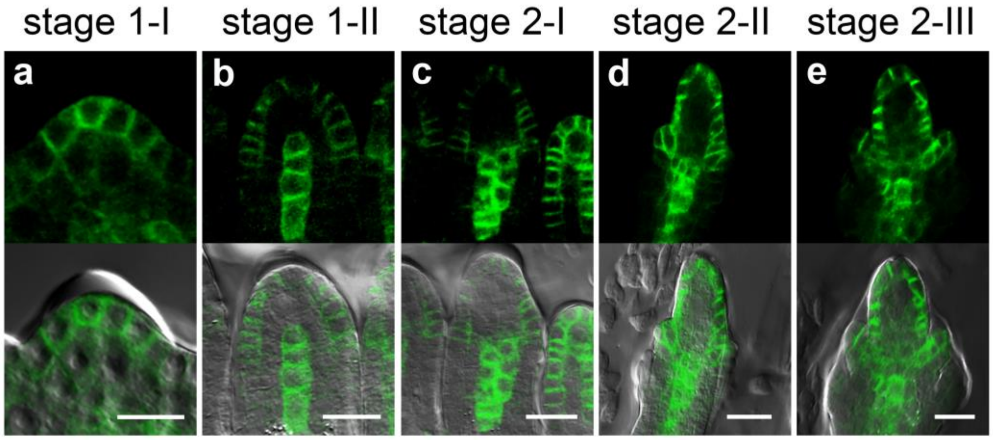

Supplementary Fig. 8 The PIN1 protein expression during MMC differentiation.

a-e, Confocal (top row) as well as merged confocal and DIC (bottom row) images showing the PIN1 protein **expression** in ovules expressing *pPIN1::PIN1-GFP*. **a**, At stage 1-I, PIN1 is mainly present in the epidermis of ovule primordium. **b**, At stage 1-II, PIN1 is in the epidermis

of nucellus and in one file of cells in the central chalaza. **c**, At stage 2-I, PIN1 is in the epidermis of nucellus and in two files of cells in the central chalaza. **d**, At stage 2-II, PIN1 is in the epidermis of nucellus, the inner integument, and the central chalaza. **e**, At stage 2-III, PIN1 is in the epidermis of nucellus, inner and outer integuments, and the central chalaza. Experiments were repeated three times with similar results. Scale bars, 10 µm.

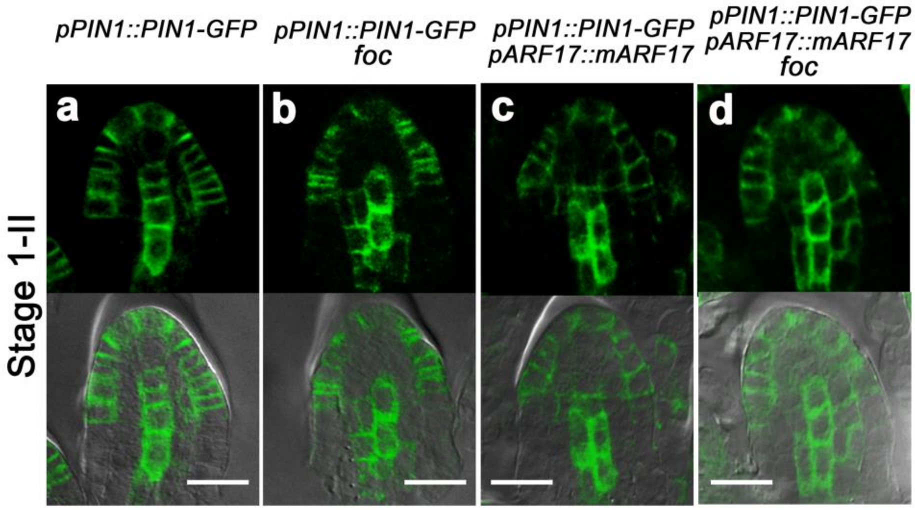

Supplementary Fig. 9 miR160 and *ARF17* are important for PIN1 expression during MMC differentiation at stage 1-II.

a-**d**, Confocal (top row) as well as merged confocal and DIC (bottom row) images showing the PIN1 protein **expression** in ovules at stage 1-II. **a**, PIN1 in the epidermis of nucellus and in one file of cells in the central chalaza of the *pPIN1::PIN1-GFP* ovule. **b**-**d**, Expanded PIN1 expression domains in two or three files of cells in the central chalaza of *pPIN1::PIN1-GFP foc* (**b**), *pPIN1::PIN1-GFP pARF17::mARF17* (**c**), and *pPIN1::PIN1-GFP pARF17::mARF17 foc* (**d**) ovules. Experiments were repeated three times with similar results. Scale bars, 10 µm.

Supplementary Table 1. Ovule and seed defects in *foc*, *mARF10*, *mARF16*, *mARF17*, and *mARF17 foc* plants

	Aborted Ovules (%)	Aborted Seeds (%)	Aborted Ovules and Seeds (%)	Normal Seeds (%)
WT	2.94±1.23	1.42±1.14	4.36±2.24	95.64±2.31
foc	66.12±4.88	21.65±4.91	87.77±8.65	12.23±5.31
mARF10	17.34±4.82	4.23±2.65	21.57±7.06	69.67±4.59
mARF16	6.03±2.51	4.48±1.82	10.51±4.16	89.49±2.87
mARF17	44.98±7.84	34.88±4.69	79.86±11.32	20.14±6.51
mARF17 foc	89.55±4.61	7.48±2.3	97.03±6.42	2.98±0.76

Supplemmentary Table 2. Constructs generated in this study

Constructs	Cloning Method	Template	Primers	Cloning Sites	Construct Backbone
pENTR-pARF10::	TOPO cloning		zp1458+zp1459		pENTR/D-TOPO
pENTR-pARF16::	TOPO cloning		zp1466+zp1467		pENTR/D-TOPO
pENTR-pARF17::	TOPO cloning		zp1475+zp1476		pENTR/D-TOPO
pENTR-pARF10 ::mARF10	Ligation	C00034	(zp1460+zp1296)+ (zp1295+zp1461)	XhoI+SpeI	pENTR/D-TOPO
pENTR-pARF16::mARF16	Ligation	U11294	(zp1468+zp1298)+ (zp1297+zp1469)	EcoRI+XbaI	pENTR/D-TOPO
pENTR-pARF17::mARF17	Ligation	C104833	(zp2410+zp1300)+ (zp1299+zp2769)	KpnI+AscI	pENTR/D-TOPO
pENTR-pARF17::ARF17		C104833	zp1477+zp1478	SmaI+XbaI	pENTR/D-TOPO
pENTR-pKNU::	TOPO cloning	genomic DNA	zp2400+zp2702		pENTR/D-TOPO
pENTR-pKNU ::mARF17	Ligation		zp2848+zp2769	NgoMIV+AscI	pENTR/D-TOPO
pENTR-pKNU ::STTM160/160-48	Ligation		zp2950+zp2948	NgoMIV+AscI	pENTR/D-TOPO
pENTR-pMIR160a5'	TOPO cloning	T16B24	zp247+zp2153		pENTR/D-TOPO
pENTR-pMIR160a5'-NSL-3xGFP	Ligation	pGreenII KAN SV40-3×GFP		KpnI+XbaI	pENTR/D-TOPO
pENTR-pMIR160a5'-NSL-3xGFP-MIR160a3'	Ligation	T16B24	zp2254+zp2255	NheI+AscI	pENTR/D-TOPO
pMIR160a5'-NSL-3xGFP- MIR160a3'	LR recombination				pGWB1
pENTR-pUBI10::NSL-3xGFP	Ligation	genomic DNA	zp2217+zp2944	SacII+KpnI	pENTR-pMIR160a5'-NSL-3xGFP
pENTR-pUBI10::miR160sens-NSL-3xGFP		genomic DNA	zp2217+zp2945	SacII+KpnI	pENTR-pMIR160a5'-NSL-3xGFP
pENTR-EMS1::	TOPO cloning	T28J14	zp91+zp591		pENTR/D-TOPO
pENTR-EMS1::YUC1	Ligation	pCHF3	zp2016+zp2326	NgoMIV+AscI	pENTR/D-TOPO
pARF10::mARF10	LR recombination				pGWB1
pARF16::mARF16	LR recombination				pGWB1
pARF17::mARF17	LR recombination				pGWB1
pARF17::ARF17-GFP					pGWB4
pARF17::mARF17-GFP	LR recombination				pGWB4
pKNU::mARF17	LR recombination				pGWB1

pKNU ::STTM160/160-48	LR recombination				pGWB1
pENTR-pUBI10::NSL-3xGFP	LR recombination				pGWB1
pENTR-pUBI10::miR160sens-NSL-3xGFP	LR recombination				pGWB1
EMS1::YUC1	LR recombination				pGWB1
crispr-arf17	Golden Gate reaction		zp2574+zp2575	BsaI	pHEE401E

Supplementary Table 3. Primers used in this study

Primers	Sequences (5' to 3')	Purposes
z+A4:C31p	CACCTATTATTCTTCGATGGTAGAAGTTT	*pARF10::*
zp1459	ACTAGTATCGATCTAGACGAAGTTGTGTAACCCC	
zp1466	CACCATAATTGGATATTGGATTTTTGTT	*pARF16::*
zp1467	TCTAGAGAATTCATTTTTTGTGACCGTTTTTGC	
zp1475	CACCTCTCACCGGAGCTGACAAAA	*pARF17::*
zp1476	TCTAGACCCGGGAGGTATTTGTTTTCAGTGTAAA	
zp1460	CACCATCGATATGGAGCAAGAGAAAAGC	*mARF10*
zp1296	AGCTTGTCGGGCCCCTTGAATCCCTGCAGGAGCATTATTGTTG	
zp1295	CTGCAGGGATTCAAGGGGCCCGACAAGCTCAACAACTCTTCGG	
zp1461	ACTAGTAGCGAAGATGCTGAGCGGAC	
zp1468	CACCGAATTCATGATAAATGTGATGAATCC	*mARF16*
zp1298	ATTATGTCTTGCCCCTTGCAAACCCACGGGAACATTGT	
zp1297	CGTGGGTTTGCAAGGGGCAAGACATAATGCTCATCAGTACTACGG	
zp1469	TCTAGATACTACAACGCTCTCACTTCCT	
zp1477	CACCCCCGGGATGTCACCGCCGTCG	*mARF17*
zp1300	ATATTGCCGTGCACCTTGCATTCCAGCAGGAAATGTAGAATACG	
zp1299	CTGCTGGAATGCAAGGTGCACGGCAATATGATTTTGGGTCT	
zp1478	TCTAGAACCTTGGGAGCTAGAACC	
zp1477	CACCCCCGGGATGTCACCGCCGTCG	*ARF17*
zp1478	TCTAGAACCTTGGGAGCTAGAACC	
zp2400	CACCGCGGCCGCCAAAGCTTTTATGGTAGATTTGTTCTG	*pKNU::*
zp2702	CACCTCTAGAGCCGGCCTCGAGTTTTGAGAGGTTCTTAAGCTACAGAG	
zp2848	GGGCCGGCATGTCACCGCCGTCGGCAACC	*mARF17*
zp2769	CTTGGCGCGCCCACCTTGGGAGCTAGAACCTGCGTTG	
zp2950	AGGCCGGCCATTTGGAGAGGACAGCCCAAG	*STTM160/160-48*
zp2948	GCGGCGCGCCCTGGTGATTTCAGCGTACCG	
zp2217	CACCCCGCGGGTCGACGAGTCAGTAATAAACG	*pUBI10::*
zp2944	AGGGTACCCTGTTAATCAGAAAAACTCAGA	
zp2945	AGGGTACCTGCCTGGCTCCCTGCATGCCACTGTTAATCAGAAAAACTC AGA	*pUBI10::miR160s ensor*
zp247	CACCGCGAATTGTGATCTGAATACAATG	*pMIR160a5'*
zp2153	GCTCTAGAGGTACCGGATGAGAGAGATACATGTGTGTATAT	
zp2254	TTAGCTAGCCTCGAGAAATTTTGGTTTCAAATGCACAATTG	*MIR160a3'*
zp2255	TTAGGCGCGCCCCGTCTTCTTGATACCAAATTAC	
zp91	CACCCAGAGAGAACCAATGCAACTC	*pEMS1::*
zp591	GGGGTACCAAGCCGGCGTTCTTTTAGAGAAGGAG	
zp2016	GCGGATCCGCCGGCATGGAGTCTCATCCTCACAACAA	*YUC1*
zp2326	GCGGCGCGCCTCAGGATTTAGAGGTAAAGACAAAACGA	
zp2574	ATTGGCACCGGCGGTTGCCGACGG	23 bp of *ARF17*
zp2575	AAACCCGTCGGCAACCGCCGGTGC	
zp2654	CCCGTAGCATCGTAACAGTAA	Sequencing for *crispr-arf17*
zp2705	GTCGGGTCTACTTCACGGTGG	
zp2770	caccCTCGAGATGGCGACTTCTCTCTTCTTCATG	RT-PCR for *SPL*
zp1214	CATCTAGATTAAAGCTTCAAGGACAAATCAATG	

zp2636	GCGGATCCGCCGGCTGATGATGATCTTCTTCTCGGAACTC	For *spl-3* identification
zp2815	CCATGCATTGGATCGTGGAAG	
zp1289	ATTTTGCCGATTTCGGAAC	
zp853	GTTGGGATGAACCAGAAGGA	qRT-PCR for *ACTIN2*
zp854	GAGGAGCCTCGGTAAGAAGA	
zp332	GCACCTGATCCAAGTCCTTC	qRT-PCR for *ARF17*
zp2907	CAGGAAGCGGACTTAAGAAG	
zp2903	ACAAAACGACGCAGGCTAAG	qRT-PCR for *PIN1*
zp2904	AGCTGGCATTTCAATGTTCC	
zp2905	AGCTCGAGCGTCAGAGAATC	qRT-PCR for *SPL*
zp2906	CTTGGGAAGCCTTGTAGCAC	
