# Supplementary material for: Specification of female germline by microRNA orchestrated auxin signaling in Arabidopsis
Source: Nat Commun. 2022 Nov 15;13:6960. doi: 10.1038/s41467-022-34723-6 (PMC9666636; doi:10.1038/s41467-022-34723-6)
Supplement: Supplementary file 3 — Reporting Summary [file 41467_2022_34723_MOESM3_ESM.pdf]

nature portfolio

Corresponding author(s): DBPR NCOMMS-21-28430B

Last updated by author(s): Oct 28, 2022

Reporting Summary

Nature Portfolio wishes to improve the reproducibility of the work that we publish. This form provides structure for consistency and transparency in reporting. For further information on Nature Portfolio policies, see our Editorial Policies and the Editorial Policy Checklist.

Statistics

For all statistical analyses, confirm that the following items are present in the figure legend, table legend, main text, or Methods section.

n/a	Confirmed	
☐	☒	The exact sample size (*n*) for each experimental group/condition, given as a discrete number and unit of measurement
☐	☒	A statement on whether measurements were taken from distinct samples or whether the same sample was measured repeatedly
☐	☒	The statistical test(s) used AND whether they are one- or two-sided<br>*Only common tests should be described solely by name; describe more complex techniques in the Methods section.*
☒	☐	A description of all covariates tested
☒	☐	A description of any assumptions or corrections, such as tests of normality and adjustment for multiple comparisons
☐	☒	A full description of the statistical parameters including central tendency (e.g. means) or other basic estimates (e.g. regression coefficient) AND variation (e.g. standard deviation) or associated estimates of uncertainty (e.g. confidence intervals)
☐	☒	For null hypothesis testing, the test statistic (e.g. F, t, r) with confidence intervals, effect sizes, degrees of freedom and P value noted<br>*Give P values as exact values whenever suitable.*
☒	☐	For Bayesian analysis, information on the choice of priors and Markov chain Monte Carlo settings
☒	☐	For hierarchical and complex designs, identification of the appropriate level for tests and full reporting of outcomes
☒	☐	Estimates of effect sizes (e.g. Cohen's *d*, Pearson's *r*), indicating how they were calculated

Our web collection on statistics for biologists contains articles on many of the points above.

Software and code

Policy information about availability of computer code

Data collection	*Provide a description of all commercial, open source and custom code used to collect the data in this study, specifying the version used OR state that no software was used.*
Data analysis	Images were processed with ImageJ and Photoshop CS6 using the same settings. qRT-PCR results were analyzed by the comparative C(T) method.

For manuscripts utilizing custom algorithms or software that are central to the research but not yet described in published literature, software must be made available to editors and reviewers. We strongly encourage code deposition in a community repository (e.g. GitHub). See the Nature Portfolio guidelines for submitting code & software for further information.

Data

Policy information about availability of data

All manuscripts must include a data availability statement. This statement should provide the following information, where applicable:

- Accession codes, unique identifiers, or web links for publicly available datasets
- A description of any restrictions on data availability
- For clinical datasets or third party data, please ensure that the statement adheres to our policy

The data supporting the findings in this study are available from the corresponding author upon reasonable request.

Field-specific reporting

Please select the one below that is the best fit for your research. If you are not sure, read the appropriate sections before making your selection.

☒ Life sciences ☐ Behavioural & social sciences ☐ Ecological, evolutionary & environmental sciences

For a reference copy of the document with all sections, see nature.com/documents/nr-reporting-summary-flat.pdf

Life sciences study design

All studies must disclose on these points even when the disclosure is negative.

Sample size	Pertinent studies from the literature were used to determine the sample size. These studies are cited in the manuscript.
Data exclusions	No data were excluded from analyses.
Replication	All experiments were performed in biological triplicates.
Randomization	Allocation of individual plants into experimental groups was random. Samples from wild-type, mutants, transgenic plants, and NPA treated plants were randomly collected.
Blinding	The investigators were not blinded to the materials in the experiment during sampling since sample collection was based on phenotypes.

Reporting for specific materials, systems and methods

We require information from authors about some types of materials, experimental systems and methods used in many studies. Here, indicate whether each material, system or method listed is relevant to your study. If you are not sure if a list item applies to your research, read the appropriate section before selecting a response.

Materials & experimental systems

n/a	Involved in the study	
☐	☒	Antibodies
☒	☐	Eukaryotic cell lines
☒	☐	Palaeontology and archaeology
☒	☐	Animals and other organisms
☒	☐	Human research participants
☒	☐	Clinical data
☒	☐	Dual use research of concern

Methods

n/a	Involved in the study	
☒	☐	ChIP-seq
☒	☐	Flow cytometry
☒	☐	MRI-based neuroimaging

Antibodies

Antibodies used	Anti-GFP antibody (Torrey Pines Biolabs; catalog no. TP401; Lot Number: 071519) was used at 1:100 dilution.<br>Goat Anti-Rabbit IgG Antibody (Alexa Fluor® 488) (Jackson ImmunoResearch Lab; 111-545-003) was used at 1:1000 dilution.
Validation	Anti-GFP antibody (Torrey Pines Biolabs; catalog no. TP401) has been validated by Torrey Pines Biolabs via Western blot. This antibody was also validated in our previous work (Huang et al, 2016), which showed its specificity to the GFP protein in Arabidopsis.
